# Clinical and Radiological Features Predicting Intervertebral Autofusion after Successful Antibiotic Therapy in Pyogenic Vertebral Osteomyelitis

**DOI:** 10.3390/diagnostics11081349

**Published:** 2021-07-27

**Authors:** Insu Seong, Eunjung Kong, Ikchan Jeon

**Affiliations:** 1 Department of Neurosurgery, Yeungnam University College of Medicine, Daegu 42415, Korea; ecis9107@ynu.ac.kr; 2 Department of Nuclear Medicine, Yeungnam University College of Medicine, Daegu 42415, Korea; kongej@yu.ac.kr

**Keywords:** spine, pyogenic, osteomyelitis, autofusion, ^18^F-FDG

## Abstract

Background: Pyogenic vertebral osteomyelitis (PVO) is a bacterial infection involving the intervertebral disc, vertebral body, and paravertebral soft tissues. Damaged intervertebral structure is a major cause of persistent back pain even after successful antibiotic therapy, which can be improved by achieving autofusion or via additional surgical fixation. In this study, we analyzed the clinical and radiological features predicting intervertebral autofusion after successful antibiotic therapy in lumbar PVO. Methods: This study was retrospectively conducted with 32 patients (20 men and 12 women) diagnosed with lumbar PVO that was completely cured with no recurrences after antibiotic therapy. They were divided into two groups with (group A, *n* = 18) and without (group B, *n* = 14) intervertebral autofusion at six-month follow-up. Differences in back pain, blood inflammatory markers, and radiological features of PVO on simultaneous ^18^F-fluorodeoxyglucose positron emission tomography/magnetic resonance imaging (^18^F-FDG-PET/MRI) of the intervertebral structure between the two groups were analyzed. Results: The mean duration of antibiotic therapy was 41.44 ± 14.21 (21–89) days. Group A showed a statistically higher erythrocyte sedimentation ratio (ESR; 59.28 ± 32.33 vs. 33.93 ± 18.76 mm/h, *p* = 0.014; normal range of ESR < 25), maximum standardized ^18^F-FDG uptake (SUV_max_; 5.56 ± 1.86 vs. 3.98 ± 1.40, *p* = 0.013), and sustained extensive edematous changes on T2-weighted fat saturation (T2FS) MRI (*p* = 0.015) immediately after successful antibiotic therapy. However, no significant differences were observed in back pain, C-reactive protein, or the distribution of ^18^F-FDG uptake/contrast enhancement on ^18^F-FDG-PET/MRI (*p* > 0.05). Conclusions: Higher ESR and SUV_max_ of the intervertebral structure and sustained extensive edematous change on T2FS MRI immediately after successful antibiotic therapy are related with subsequent intervertebral autofusion, which should be carefully considered when assessing therapeutic response in PVO.

## 1. Introduction

Pyogenic vertebral osteomyelitis (PVO) is a bacterial infection involving the spine and adjacent structures [1]. PVO shows non-specific symptoms, which are not necessarily accompanied with a fever. In addition, the imaging findings do not always correlate with symptoms [2,3,4,5]. Moreover, the causative bacteria are not identified in approximately 50% of patients with PVO. Hence, they are usually treated with an average of about six weeks of empirical parenteral antibiotics [2,6]. Unfortunately, no clear treatment guidelines have been established because of the regional variability of causative microorganisms and resistance to antibiotics [7]. The therapeutic response to antibiotic therapy for PVO has been assessed based on clinical symptoms and blood inflammatory markers. However, clinical symptoms can vary between individuals, and blood inflammatory markers, including erythrocyte sedimentation ratio (ESR) and C-reactive protein (CRP), are easily affected by the other physical conditions [4]. In addition, magnetic resonance imaging (MRI), recognized as the best imaging modality for spinal disease, is not helpful in determining therapeutic response of PVO. MRI cannot accurately distinguish the residual PVO lesions from the structural damage or the tissues restored after successful antibiotic therapy [8].

Recently, it has been reported that simultaneous ^18^F-fluorodeoxyglucose positron emission tomography (^18^F-FDG-PET) can be used to determine therapeutic response in spinal infections. ^18^F-FDG-PET is less affected by other physical conditions and shows more objective results compared with blood inflammatory markers [9]. Several studies have shown a higher diagnostic accuracy in detecting residual infectious lesion by measuring the degree of ^18^F-FDG uptake on ^18^F-FDG-PET than by using CRP and MRI [9,10,11]. In addition, Jeon et al. [5] reported differences between the degree of ^18^F-FDG uptake and clinical symptoms in patients with cured PVO depending on the site of major structural damage. Therefore, additional studies are required to assess therapeutic response more accurately. PVO mainly destroys the intervertebral structure, which can become unstable and result in sustained back pain even after successful antibiotic therapy. Usually, the damaged intervertebral structures are stabilized with autofusion between the vertebrae; however, some patients showing sustained instability require additional surgical fixation.

In this study, we analyzed the clinical and radiological features identified at the time of discontinuation of antibiotic therapy as it relates to intervertebral autofusion at six-month follow-up for patients with cured PVO.

## 2. Materials and Methods

### 2.1. Patients and Data Collection

This retrospective study included 61 patients (37 men and 24 women) with lumbar PVO. It was conducted using prospectively collected clinical and radiological data from February 2017 to September 2020 in a single tertiary hospital. Diagnosis of lumbar PVO was made mainly based on the clinical symptoms (including fever, back pain, or neurological signs) and specific MRI findings with a contiguous single PVO lesion; the diagnosis was not dependent on the causative microorganism identified from PVO lesion or blood cultures. Patients with tuberculous vertebral osteomyelitis, tumor, bone infection other than the PVO, trauma, concomitant severe medical problems, spinal instrumentation or bone cement on the PVO lesion, recurrence of PVO within 6-month follow-up, pregnancy, or those < 20 years old were excluded. The PVO lesion comprised the upper and lower vertebrae centering on the infected disc with or without involving paraspinal muscle; such lesions were defined as single level PVO [10]. Those comprising three vertebrae involving two infected discs were defined as two levels of PVO.

All patients provided voluntary written informed consent for performing additional simultaneous ^18^F-FDG-PET/MRI during the clinical assessment for assessing therapeutic response. The entire study and the analysis of clinical and radiological data were conducted under the approval of the institutional review board (Yeungnam University Hospital, 2016-12-019-013, and 22 December 2016).

### 2.2. Clinical Assessment

All patients underwent clinical assessments for therapeutic response based on the clinical symptoms and blood inflammatory markers after conducting parenteral antibiotic therapy for at least 3 weeks. The parenteral antibiotics to be used were selected after consulting infectious disease physicians. Back pain was measured using visual analog scale (VAS) with 0 representing no pain to 10 representing maximum pain. ESR (normal range of <25 mm/h) and CRP (normal range of <0.5 mg/dL) were used as the blood inflammatory markers.

Cure was defined as improved clinical symptoms with no fever and normalized CRP for at least six months after the discontinuation of parenteral antibiotic therapy. Recurrence was defined as re-developing clinical symptoms with/without fever, re-elevation of CRP, or newly identified or aggravated PVO on MRI within six months of follow-up period.

### 2.3. Radiological Criteria of Intervertebral Autofusion

The intervertebral fusion was determined based on the criteria presented by Lee et al. [12]:

(1) Definitive fusion: Definitive bony trabecular bridging across the graft/host interface, no motion (less than 3°) on flexion–extension radiographs, and no gap at the interface.
(2) Probable fusion: No definitive bony trabecular crossing, no detectable motion, and no identifiable gap at the interface.
(3) Possible pseudoarthrosis: No bony trabecular crossing, no motion, but an identifiable gap at the interface.
(4) Definite pseudoarthrosis: No traversing trabecular bone, definitive gap, and motion greater than 3°.

We categorized the patients into two groups depending on the intervertebral autofusion at six-month follow-up. Group A, which included above categories 1 and/or 2, was defined as intervertebral autofusion, while group B, which included above categories 3 and/or 4, was defined as no intervertebral autofusion.

### 2.4. Radiological Assessment of ^18^F-FDG-PET/MRI

In the patients with cured PVO and no recurrence, ^18^F-FDG-PET/MRIs obtained when parenteral antibiotic therapy was discontinued were included in this study. Two physicians with ≥10 years of experience in nuclear medicine analyzed the imaging data obtained from ^18^F-FDG-PET/MRI independently with information about the clinical status of the patients.

#### 2.4.1. Intensity of ^18^F-FDG Uptake on the Intervertebral Structure of Cured PVO Lesion 

The maximum standardized ^18^F-FDG uptake value (SUV_max_) on the intervertebral structures, including the disc and endplates of the PVO lesion, was evaluated on ^18^F-FDG-PET/MRI. The vertebral endplates are thin structures located above and below the ends of the intervertebral disc and mechanical interface between the vertebral body and the intervertebral disc made of osseous and cartilaginous components [13,14]. The SUV_max_ values were determined using a computerized imaging system, and the difference in the SUV_max_ values between the two groups was analyzed.

#### 2.4.2. Distribution Patterns of ^18^F-FDG Uptake, Contrast Enhancement, and Edematous Change on ^18^F-FDG-PET/MRI of Cured PVO Lesion

The distribution pattern of ^18^F-FDG uptake on ^18^F-FDG-PET, contrast enhancement on T1-weighted contrast (T1C) MRI, and high signal intensity implying edematous changes on T2-weighted fat saturation (T2FS) MRI in the PVO lesion were interpreted based on the criteria presented by Yu et al. [4]. In case of any disagreement on the distribution pattern between the two nuclear medicine physicians, the final decision was reached after a discussion. The distribution patterns were graded as follows.

(1) Grade I: Activities on the overall vertebral body, paravertebral soft tissue/muscle, and epidural space with lower or similar intensity compared to the normal region.
(2) Grade II: Limited activities on the margin or bulk of a destroyed disc/endplate rather than the vertebral body, paravertebral soft tissue/muscle, and epidural space with higher intensity than the normal region.
(3) Grade III: Significantly increased activities compared to the normal region on the overall vertebral body, paravertebral soft tissue/muscle, and epidural space.

### 2.5. Data Acquisition of PET/MRI

Prior to injecting FDG (3.7 MBq/kg) to perform simultaneous ^18^F-FDG-PET/MRI (Biograph mMR; Siemens Healthcare, Erlangen, Germany), the patients were asked to fast for at least 6 h and their blood glucose levels were maintained at <8.9 mmol/L. Data acquisition of ^18^F-FDG-PET/MRI was initiated 60 min after the injection of FDG. The patient was placed and scanned in one–two bed positions under the approved surface coil. Data acquisition of ^18^F-FDG-PET was performed for >20 min; the MRI data were simultaneously acquired using a preset sequence protocol [10].

### 2.6. Statistical Analysis

A Chi-squared test was performed to assess the relationship between the categorical variables. Student’s *t*-test for parametric continuous variables and Mann–Whitney U test for non-parametric continuous variables were used to compare the two population means. Statistical analyses were performed with SPSS version 25.0 (SPSS Inc., Chicago, IL, USA); *p* values < 0.05 were considered statistically significant.

## 3. Results

### 3.1. Demographic and Clinical Data

Among the 61 patients, 29 were excluded because of the follow-up loss (*n* = 2), bone infection other than the PVO (*n* = 1), concomitant severe medical problems (*n* = 3), spinal instrumentation or bone cement on the PVO lesion (*n* = 7), recurrence during the follow-up period (*n* = 2), and no radiography at 6-month follow-up (*n* = 14). The final analyses were performed on 32 patients (20 men and 12 women) with a mean age of 69.25 ± 10.20 (50–85) years. They were divided into two groups depending on whether autofusion was noted at six-month follow-up radiographs (18 of group A: intervertebral autofusion, 14 of group B: no intervertebral autofusion). No statistically significant differences were noted for age, sex, initial extent of PVO, initial involvement of PVO, and initial clinical symptoms between the two groups (*p* > 0.05). The cause of PVO was identified as spontaneous and procedure-related in 43.7% (14/32) and 56.3% (18/32) of the patients, respectively (*p* > 0.05). The mean follow-up period was 12.84 ± 6.98 (6–35) months. Detailed data are presented in Table 1.

### 3.2. Microorganisms and Antibiotics

The identification rate of causative microorganism was 40.6% (13/32) in blood or tissue culture. Culture for PVO lesion was the major route to identify the causative microorganism (61.5%, 8/13). Methicillin-sensitive *Staphylococcus aureus* (MSSA) was the main causative microorganism (38.5%, 5/13). The mean duration of the parenteral antibiotic therapy was 41.44 ± 14.21 (21–89) days. Group A showed a statistically significant longer duration of parenteral antibiotic therapy compared with group B (45.94 ± 16.09 vs. 35.64 ± 8.86 days, *p* = 0.040). Detailed data are presented in Table 2.

### 3.3. Clinical Features

Blood inflammatory markers including ESR and CRP were measured at initial diagnosis and discontinuation of antibiotic therapy, respectively. CRP showed no statistically significant difference at initial diagnosis (8.97 ± 7.91 vs. 8.79 ± 9.31 mg/dL, *p* = 0.722) and discontinuation of antibiotic therapy (0.85 ± 1.14 vs. 0.73 ± 0.78 mg/dL, *p* = 0.635) between the two groups, respectively. However, group A showed statistically significant higher ESR compared with that shown by group B at initial diagnosis (74.67 ± 23.57 vs. 50.21 ± 29.94 mm/h, *p* = 0.015) and discontinuation of antibiotic therapy (59.28 ± 32.33 vs. 33.93 ± 18.76 mm/h, *p* = 0.014). Back pain was measured using VAS at initial diagnosis and discontinuation of antibiotic therapy. No statistically significant difference was observed at initial diagnosis (7.94 ± 0.64 vs. 7.79 ± 1.31, *p* = 0.985) and discontinuation of antibiotic therapy (4.39 ± 0.92 vs. 4.29 ± 1.14, *p* = 0.955) between the two groups. Detailed data are presented in Table 3.

### 3.4. Radiological Features

The locations of SUV_max_ on ^18^F-FDG-PET were identified as 26 on intervertebral structures and 6 on vertebral bodies/paraspinal muscles. No statistically significant difference was noted in the location of SUV_max_ between the two groups. The mean SUV_max_ of intervertebral structure was 4.87 ± 1.83 (2.10–10.51) in all patients. The difference in the mean SUV_max_ values of the two groups was statistically significant (5.56 ± 1.86 vs. 3.98 ± 1.40, *p* = 0.013). Detailed data are presented in Table 3.

The distribution patterns of the ^18^F-FDG uptake on ^18^F-FDG-PET, contrast enhancement on T1C MRI, and high signal intensity on T2FS MRI in the PVO lesions of all patients were examined. There were 18 of grade II and 14 of grade III on ^18^F-FDG-PET; 4 of grade II and 28 of grade III on T1C MRI; and 9 of grade II and 23 of grade III on T2FS MRI. A statistically significant difference was noted in the distribution pattern of high signal intensity on T2FS MRI between the two groups (*p* = 0.015). However, no statistically significant difference was observed in the distribution patterns of the ^18^F-FDG uptake on ^18^F-FDG-PET (*p* = 0.419) and contrast enhancement on T1C MRI (*p* = 0.178). Detailed data are presented in Table 4.

## 4. Discussion

Jeon et al. [10] reported the usefulness of ^18^F-FDG-PET for assessing the therapeutic response in PVO. They observed that ^18^F-FDG-PET showed better diagnostic accuracy for detecting residual lesions than CRP or MRI. They also showed that the combination of FDG-PET and CRP can be the best modality. ^18^F-FDG-PET is based on the ^18^F-FDG uptake by the activated inflammatory cells consuming a large amount of glucose during phagocytoses. Conversely, the ^18^F-FDG uptake can decrease because of the disappearance of activated inflammatory cells after successful antibiotic therapy. The ^18^F-FDG uptake on the PVO lesion is less affected by other conditions and can be used as an independent modality for assessing the therapeutic response in PVO [15,16,17]. In the distribution pattern of the ^18^F-FDG uptake on the PVO lesion, the cured PVO lesion shows that the ^18^F-FDG uptake is limited to the intervertebral structure in contrast to the extensive distribution of the ^18^F-FDG uptake observed in the non-cured PVO lesion [5,10]. However, the clinical and radiological features of the cured PVO vary depending on the location of the major structural damage of the PVO lesion. When an intervertebral structure is involved, it may lead to more severe back pain and a higher CRP than those involved in vertebral body/paravertebral muscle, showing favorable clinical features despite more advanced structural damage with a higher ^18^F-FDG uptake [5]. These findings may be helpful for assessing the therapeutic response in patients with PVO presenting various structural damages and clinical symptoms.

**Figure 1:**
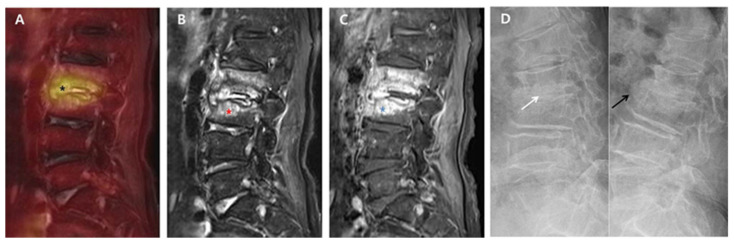
^18^F-FDG-PET/MRI immediately after successful antibiotic therapy and radiographs at six-month follow-up in a patient with autofusion (group A). A 78-year-old female patient shows PVO of L2–3 on ^18^F-FDG-PET/MRI with SUV_max_ 5.03 of the intervertebral structure (black asterisk) after 56 days of ceftriaxone (ESR 71 mm/h and CRP 0.67 mg/dL). In the distribution pattern of the PVO lesion, ^18^F-FDG uptake (black asterisk) on ^18^F-FDG-PET (**A**) is limited on the damaged disc and endplates (grade II). However, T2FS MRI (**B**) and T1C MRI (**C**) show significantly increased high signal intensity (red asterisk) and contrast enhancement (blue asterisk) on the overall PVO lesion (grade III), respectively. On the dynamic radiographs at six-month follow-up (**D**), stable intervertebral structure presenting with the formation of syndesmophyte (black arrow) is noted with no intervertebral gap (white arrow). ^18.^ F-FDG-PET/MRI, ^18^F-fluorodeoxyglucose positron emission tomography/magnetic resonance imaging; SUV_max_, maximum standardized ^18^F-FDG uptake value; PVO, pyogenic vertebral osteomyelitis; CRP, C-reactive protein (normal range of < 0.5 mg/dL); ESR, erythrocyte sedimentation rate (normal range of <25 mm/h); T2FS, T2-weighted fat saturation; T1C, T1-weighted contrast; MRI, magnetic resonance imaging.

**Figure 2:**
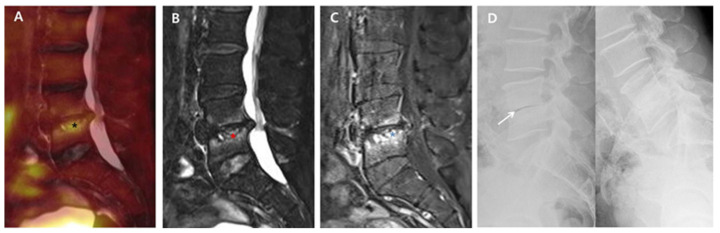
^18^F-FDG-PET/MRI immediately after successful antibiotic therapy and radiographs at six-month follow-up in a patient with no autofusion (group B). A 73-year-old male patient shows PVO of L4–5 on ^18^F-FDG-PET/MRI with SUV_max_ 3.83 of the intervertebral structure (black asterisk) after 56 days of ceftriaxone (ESR 37 mm/h and CRP 0.16 mg/dL). In the distribution pattern of the PVO lesion, ^18^F-FDG uptake (black asterisk) on ^18^F-FDG-PET (**A**), high signal intensity (red asterisk) on T2FS MRI (**B**), and contrast enhancement (black asterisk) on T1C MRI (**C**) are limited around the destroyed disc and endplates rather than the bone and soft tissue with almost improved discitis (grade II). The dynamic radiographs at six-month follow-up (**D**) show an unstable intervertebral structure presenting with an intervertebral gap (white arrow), and there is no definite formation of syndesmophyte. ^18.^ F-FDG-PET/MRI, ^18^F-fluorodeoxyglucose positron emission tomography/magnetic resonance imaging; SUV_max_, maximum standardized ^18^F-FDG uptake value; PVO, pyogenic vertebral osteomyelitis; CRP, C-reactive protein (normal range of <0.5 mg/dL); ESR, erythrocyte sedimentation rate (normal range of <25 mm/h); T2FS, T2-weighted fat saturation; T1C, T1-weighted contrast; MRI, magnetic resonance imaging.

Back pain caused by the destruction of intervertebral discs and endplates may continue during the post-treatment course of PVO even after successful antibiotic therapy. Although some PVO lesions presenting back pain related with sustained instability may require additional fusion surgery, this study showed that most PVO lesions were stabilized with intervertebral autofusion or maintained joint function without any instability after successful antibiotic therapy. At 6-month follow-up, intervertebral autofusion was achieved in 59.3% of patients, and instability was identified in only 6.2% (2/32) of patients. However, there is still insufficient literature on the rate of autofusion or instability requiring surgical treatment. In this study, 14 patients were excluded because of insufficient radiological data at six-month follow-up, which can be a major limitation in obtaining higher levels of evidence; however, no patient was treated with additional fusion surgery. Hence, we can suggest that a large portion of intervertebral damage caused by PVO can be treated with intervertebral autofusion or preserved as an intervertebral joint after successful antibiotic therapy. Compared to group B with no intervertebral autofusion at six-month follow-up, group A with intervertebral autofusion showed more extensive edematous changes to vertebral bodies and a higher ^18^F-FDG uptake on the intervertebral structure immediately after antibiotic therapy (Figure 1 and Figure 2).

We investigated pathophysiological characteristics related to the specific radiological features identified in group A. The ^18^F-FDG uptake in a vertebral fracture has been reported previously. He et al. [18] reported SUV_max_ of 1.7–4.9 with a mean value of 2.9 ± 1.0 in 18 benign vertebral compression fractures. A benign fracture may continue to show ^18^F-FDG uptake up to six weeks after the fracture. Additionally, there may be significant differences in the duration and intensity of ^18^F-FDG uptake depending on the fracture site and severity [19]. However, Jeon et al. [5] have reported sustained elevation of ^18^F-FDG uptake (SUV_max_ 4.34 ± 1.24) in the intervertebral structure of PVO immediately after successful antibiotic therapy with a mean duration of approximately 40 days. This phenomenon was observed mainly because of the formation of vascular structures and chronic inflammation presenting as part of the healing process of the destroyed structures after acute inflammation against infection [8,9]. In addition, we believe that the degree of structural damage caused by PVO is greater than that observed in a benign fracture, which may account for the higher ^18^F-FDG uptake in PVO. Increased ^18^F-FDG uptake on the intervertebral structure is also correlated with the occurrence and magnitude of endplate subsidence [20]. Suto et al. [21] reported that a higher ^18^F-FDG uptake and ESR are related to the disease activity and severity of joint destruction in rheumatoid arthritis. We noted higher ^18^F-FDG uptake in group A than in group B because of the profound damage of the intervertebral structure at initial diagnosis and aggravation during the treatment period. In particular, a higher ESR at initial diagnosis in group A supports the presence of more severe structural destruction at initial diagnosis than that in group B.

Intervertebral autofusion observed in group A is usually characterized by the formation of syndesmophytes at the anterior portion of intervertebral structure as in ankylosing spondylitis (AS). Although AS is not directly comparable with PVO because of their different developmental origin, AS can help in understanding the radiological features of group A because a large number of patients with AS show preceding bone marrow edema before the formation of syndesmophytes. Current studies suggest that spinal inflammation caused by tissue repair may further stimulate syndesmophyte formation or calcification [22,23]. In AS, the bridging of the intervertebral space by bony spurs or osteophytes, which emerge from the periosteum close to the joint or intervertebral space, is based on endochondral ossification. This phenomenon can cause the deposition of the chondrogenic matrix, followed by remodeling into bone, and may present a type of joint repair strategy [24]. Stress on the joint may be responsible for the formation of osteophytes; both mechanical and inflammatory stress can precipitate their formation. Formation of osteophytes can be interpreted as an attempt by the body to repair or stabilize and reduce motion in the affected joint via bone ankylosis and complete stabilization of the joint [24]. Based on the preceding bone marrow edema and subsequent ankylosis in AS, the greater mechanical and inflammatory stress in PVO compared with that in AS, which was particularly evident in group A, may lead to extensive bone marrow edema and bone bridging in a relatively short period.

The severity of damage on the intervertebral structure depends on the destructive status at initial diagnosis and its aggravation during the treatment period. MRI can reveal structural damage in the form of increased water signals, which are associated with inflammatory edema and correlated with the histopathological grading of inflammation [25,26]. Even after successful antibiotic therapy, the damaged intervertebral structure of the cured PVO is under the inflammatory repair process and exposed to mechanical stress by patient activities, which present as sustained bone marrow edema and elevated ^18^F-FDG uptake on the intervertebral structure and vertebral bodies. These issues may vary depending on the severity of structural damage and the activity of the repair processes. Therefore, extensive bone marrow edema, which can also be presented as grade III and higher ^18^F-FDG uptake on the intervertebral structure, were more frequent in group A than in group B. We believe that the higher ESR immediately after successful antibiotic therapy in group A is also responsible for the sustained elevation of ^18^F-FDG and extensive bone marrow edema. These features (sustained elevation of ESR, extensive edematous changes, and higher ^18^F-FDG uptake in the cured PVO) are suggestive of intervertebral autofusion. Therefore, it is important not to diagnose these features as a false-positive related to residual PVO in the assessment of therapeutic response.

In this study, we used ^18^F-FDG-PET/MRI for assessing the therapeutic response in PVO and attempted to identify the factors responsible for intervertebral autofusion based on the clinical and radiological features of the cured PVO. Although our study is a novel attempt using ^18^F-FDG-PET/MRI, it has some limitations as well. First, to date, ^18^F-fluoride-PET imaging is used as an indicator of osteoblastic activity, which can be more useful than ^18^F-FDG-PET to assess fracture healing [27]. However, we evaluated autofusion using ^18^F-FDG-PET, which was taken while assessing the therapeutic response in PVO, because it is effective and inevitable for assessing such therapeutic response. Second, we used radiographs to identify intervertebral autofusion and stabilization at six-month follow-up. However, computed tomography (CT) scans can afford more accurate imaging to identify the bone structure, which is a more useful modality to evaluate intervertebral fusion. Further studies with ^18^F-fluoride-PET and CT scan under a prospective study design with a large number of participants are required to identify the exact status of intervertebral autofusion after successful antibiotic therapy in PVO.

## 5. Conclusions

Clinical and radiological features of the cured PVO vary immediately after successful antibiotic therapy. Sustained elevation of ESR, extensive edematous changes on T2FS MRI, and higher ^18^F-FDG uptake on ^18^F-FDG-PET in the cured PVO imply intervertebral autofusion as the stabilizing process of the damaged intervertebral structure, which may be helpful for assessing therapeutic response in PVO.

## Figures and Tables

**Table 1:** Demographic and clinical data of 32 patients.

Factors	Group A (*n* = 18)	Group B (*n* = 14)	Total (*n* = 32)	*^#^p* Value
Age (years)	68.94 ± 10.29 (50–85)	69.64 ± 10.46 (51–85)	69.25 ± 10.20 (50–85)	0.851
Sex (Male/Female)	12/6	8/6	20/12	0.581
Initial extent of PVO (levels)	1.22 ± 0.55 (1–3)	1.21 ± 0.80 (1–4)	1.21 ± 0.64 (1–4)	0.974
Initial involvement of PVO				
Disc	12/18 (66.7%)	7/14 (50.0%)	19/32 (59.4%)	0.473
Vertebral body	10/18 (55.6%)	5/14 (35.7%)	15/32 (46.9%)	0.308
Epidural space	12/18 (66.7%)	7/14 (50.0%)	19/32 (59.4%)	0.473
Paraspinal soft tissue	16/18 (88.9%)	13/14 (92.9%)	29/32 (90.6%)	1.000
Psoas muscle	9/18 (50.0%)	4/14 (28.6%)	13/32 (40.6%)	0.289
Cause of PVO				0.283
Spontaneous	6/18 (33.3%)	8/14 (57.1%)	14/32 (43.8%)	
Procedure-related	12/18 (66.7%)	6/14 (42.9%)	18/32 (56.2%)	
Injection or acupuncture	9/12 (75.0%)	5/6 (83.3%)	14/18 (77.8%)	
Operation	3/12 (25.0%)	1/6 (16.7%)	4/18 (22.2%)	
Initial clinical symptoms				
Fever	10 18 (66.7%)	6/14 (50.0%)	16/32 (59.4%)	0.476
Back pain	18/18 (66.7%)	13/14 (50.0%)	31/32 (59.4%)	0.249
Neurologic deficit				
Radiculopathy	11/18 (61.1%)	7/14 (50.0%)	18/32 (56.2%)	0.530
Weakness	5/18 (27.8%)	1/14 (7.1%)	6/32 (18.8%)	0.138
Bowel & bladder symptoms	1/18 (5.6%)	0/14 (0.0%)	0/32 (0.0%)	0.370
Duration of follow-up (months)	12.94 ± 7.19 (6–35)	12.71 ± 6.97 (6–30)	12.84 ± 6.98 (6–35)	0.928

PVO, pyogenic vertebral osteomyelitis; *p* values of <0.05 were considered statistically significant; ***^#^*** *p* value between groups A and B.

**Table 2:** Microorganisms and antibiotics.

Factors	Values
Identification of causative microorganisms	13/32 (40.6%)
MSSA	5
MRSA	2
*Enterococcus species*	2
MRSE	1
*Streptococcus species*	3
Non	19
Diagnosis of causative microorganisms	
Blood	4/13 (30.8%)
PVO lesion	8/13 (61.5%)
Blood & PVO lesion	1/13 (7.7%)
* Duration of intravenous antibiotics (days)	
Group A	45.94 ± 16.09 (25–89)
Group B	35.64 ± 8.86 (21–48)
Total	41.44 ± 14.21 (21–89)

MSSA, methicillin-sensitive staphylococcus aureus; MRSA, methicillin-resistant staphylococcus aureus; MRSE methicillin-resistant staphylococcus epidermidis; PVO pyogenic vertebral osteomyelitis; * *p* = 0.040 between group A and B.

**Table 3:** Comparison of clinical and radiological features between groups A and B.

Factors	Group A (*n* = 18)	Group B (*n* = 14)	Total (*n* = 32)	*^#^p* Value
Initial diagnosis				
^*^ESR (mm/h)	74.67 ± 23.57 (6–109)	50.21 ± 29.94 (26–110)	63.97 ± 28.85 (6–110)	0.015
CRP (mg/dL)	8.97 ± 7.91 (0.03–24.97)	8.79 ± 9.31 (0.59–26.05)	8.89 ± 8.41 (0.03–26.05)	0.722
VAS score of back pain	7.94 ± 0.64 (5–9)	7.79 ± 1.31 (6–9)	7.88 ± 0.98 (5–9)	0.985
Discontinuation of antibiotic therapy				
^*^ESR (mm/h)	59.28 ± 32.33 (8–67)	33.93 ± 18.76 (9–120)	48.19 ± 29.74 (8–120)	0.014
CRP (mg/dL)	0.85 ± 1.14 (0.02–2.13)	0.73 ± 0.78 (0.07–5.10)	0.79 ± 0.98 (0.02–5.10)	0.635
VAS score of back pain	4.39 ± 0.92 (2–6)	4.29 ± 1.14 (3–7)	4.34 ± 1.00 (2–7)	0.955
Location of SUV_max_ Intervertebral structure Vertebral body and paravertebral muscle	16/18 (88.9%) 2/18 (11.1%)	10/14 (71.4%) 4/14 (28.6%)	26/32 (81.3%) 6/32 (18.7%)	0.209
^*^SUV_max_ of intervertebral structure	5.56 ± 1.86 (2.74–10.51)	3.98 ± 1.40 (2.10–5.98)	4.87 ± 1.83 (2.10–10.51)	0.013

PVO, pyogenic vertebral osteomyelitis; ESR, erythrocyte sedimentation rate; CRP, C-reactive protein; VAS, visual analogue scale; SUV_max_, maximum standardized ^18^F-FDG uptake value; *p* values of < 0.05 were considered statistically significant; *p* values of < 0.05 were considered statistically significant; Data sets of all values with statistical significance showed normal distributions in normality test; ^*^ Statistical significant difference between groups A and B; ***^#^*** *p* value between groups A and B.

**Table 4:** Distribution patterns of ^18^F-FDG uptake on ^18^F-FDG-PET, contrast enhancement on T1C, and high signal intensity on T2FS.

Factors	Groups	Grade I	Grade II	Grade III	Total
^18^F-FDG uptake on ^18^F-FDG-PET (*p* = 0.419)	Group A	0	9	9	18
	Group B	0	9	5	14
	Total	0	18	14	32
Contrast enhancement on T1C MRI (*p* = 0.178)	Group A	0	1	17	18
	Group B	0	3	11	14
	Total	0	4	28	32
^*^High signal on T2FS MRI (*p* = 0.015)	Group A	0	2	16	18
	Group B	0	7	7	14
	Total	0	9	23	32

^18^F-FDG-PET, ^18^F-fluorodeoxyglucose positron emission tomography; TIC, T1-weighted contrast magnetic resonance imaging; T2FS, T2-weighted fat saturation magnetic resonance imaging; MRI, magnetic resonance imaging; *p* values of < 0.05 were considered statistically significant; ^*^ Statistically significant difference between groups A and B.

## Data Availability

The datasets acquired and analyzed during the current study are available from the corresponding author on the reasonable request.
